# Impairment of GABA transporter GAT-1 terminates cortical recurrent network activity via enhanced phasic inhibition

**DOI:** 10.3389/fncir.2013.00141

**Published:** 2013-09-11

**Authors:** Daniel S. Razik, David J. Hawellek, Bernd Antkowiak, Harald Hentschke

**Affiliations:** ^1^Experimental Anesthesiology Section, Department of Anesthesiology, University Hospital of TübingenTübingen, Germany; ^2^Center for Neural Science, New York UniversityNew York, NY, USA

**Keywords:** GABA transporter, GABA reuptake, GABA receptor, NO-711, SNAP-5114, spillover, phasic inhibition, tonic inhibition

## Abstract

In the central nervous system, GABA transporters (GATs) very efficiently clear synaptically released GABA from the extracellular space, and thus exert a tight control on GABAergic inhibition. In neocortex, GABAergic inhibition is heavily recruited during recurrent phases of spontaneous action potential activity which alternate with neuronally quiet periods. Therefore, such activity should be quite sensitive to minute alterations of GAT function. Here, we explored the effects of a gradual impairment of GAT-1 and GAT-2/3 on spontaneous recurrent network activity – termed network bursts and silent periods – in organotypic slice cultures of rat neocortex. The GAT-1 specific antagonist NO-711 depressed activity already at nanomolar concentrations (IC_50_ for depression of spontaneous multiunit firing rate of 42 nM), reaching a level of 80% at 500–1000 nM. By contrast, the GAT-2/3 preferring antagonist SNAP-5114 had weaker and less consistent effects. Several lines of evidence pointed toward an enhancement of phasic GABAergic inhibition as the dominant activity-depressing mechanism: network bursts were drastically shortened, phasic GABAergic currents decayed slower, and neuronal excitability during ongoing activity was diminished. In silent periods, NO-711 had little effect on neuronal excitability or membrane resistance, quite in contrast to the effects of muscimol, a GABA mimetic which activates GABA_A_ receptors tonically. Our results suggest that an enhancement of phasic GABAergic inhibition efficiently curtails cortical recurrent activity and may mediate antiepileptic effects of therapeutically relevant concentrations of GAT-1 antagonists.

## INTRODUCTION

In large parts of the central nervous system, GABAergic inhibition counterbalances excitation and shapes neuronal activity. GABA transporters (GATs) play an integral part in GABAergic inhibition: bound to the membranes of neurons and glial cells, they remove synaptically released GABA from the extracellular space. Thus, GATs shape phasic inhibitory currents and curb or prevent spillover, the diffusion of transmitter molecules from the release site to synaptic receptors apposed to neighboring release sites under the same synaptic bouton, to peri- and extrasynaptic receptors or even to receptors at neighboring synapses. In addition, as transport of GABA is determined by electrochemical gradients, it may revert, and thus establishes submicromolar concentrations of ambient GABA (Attwell et al., 1993; Wu et al., 2003, 2007). Three major subtypes of GAT are expressed in rodent brain. In neocortex, GAT-1 is expressed at a high density and in vicinity to synaptic GABA release sites, mostly in presynaptic boutons of interneurons and astroglial processes (Minelli et al., 1995; Chiu et al., 2002), whereas GAT-3 and at weaker expression, GAT-2, are also found in neuronal and glial compartments which are more remote from synapses (Minelli et al., 1996).

Downregulation of GATs can lead to spillover of synaptically released GABA and a boost of inhibition (Thompson and Gähwiler, 1992; Jensen et al., 2003; Keros and Hablitz, 2005; Bragina et al., 2008; Gonzalez-Burgos et al., 2009; Song et al., 2013). Although both phasic and tonic inhibition can be enhanced, it is currently unresolved which mode of inhibition would gain most in impact in cortical networks with self-sustained, recurrent action potential (AP) activity. During sparse neuronal activity, such as in cortical Down states, GATs are not critical for the clearance of the small quantities of synaptically released GABA (Thompson and Gähwiler, 1992; Bragina et al., 2008; Gonzalez-Burgos et al., 2009), which supposedly diffuse out of the synaptic cleft without activating synaptic receptors repeatedly (Mozrzymas, 2004). Consequently, in such phases, impairment of GATs should not have an impact on phasic GABAergic inhibition, but should slow down the equilibration of cytosolic and ambient [GABA], possibly reducing tonic currents (Kinney, 2005). By contrast, during synchronous and repetitive activation of numerous GABAergic synapses via electrical stimulation, impairment of GATs was found to induce a massive spillover of transmitter and synaptic crosstalk (Thompson and Gähwiler, 1992; Jackson et al., 1999; Overstreet and Westbrook, 2003; Keros and Hablitz, 2005; Gonzalez-Burgos et al., 2009). In conditions between such extremes, GATs may subserve a dual role, quickly switching between removing an excess of ambient GABA and contributing to slow phasic inhibition (Gaspary et al., 1998; Wu et al., 2007; Ransom et al., 2013). As it is unclear which direction of transport prevails in various phases of recurrent AP activity, it is difficult to predict the net effects of GAT impairment. The fact that GAT impairment alters the degree and pattern of recurrent activity (Thompson and Gähwiler, 1992; Pfeiffer et al., 1996) exacerbates the uncertainty.

The current study was motivated by the hypothesis that an impairment of GATs, particularly of GAT-1, should primarily accentuate phasic inhibition. We base our hypothesis on the concept of negative feedback in cortical networks: diminished uptake of GABA close to the synaptic release sites should transiently enhance currents through synaptic and perisynaptic GABA_A_ receptors. This, in turn, should rapidly curb AP activity, that is, synaptic GABA release, and thus minimize a major source of ambient GABA required for tonic currents in cortical networks (Glykys and Mody, 2007b). To test this hypothesis, we used organotypic slice cultures of neocortex. These preparations are characterized by a high degree of synaptic connectivity and recurrent phases of intense AP activity interleaved with phases of neuronal quiescence (Crain and Bornstein, 1964; Johnson and Buonomano, 2007), characteristics which make them ideally suited to the current investigation. We found that GAT-1 impairment did indeed curtail recurrent activity bursts by enhancing phasic inhibition, and led to a strong depression of activity, whereas GAT-2/3 impairment was much less effective in depressing activity.

## MATERIALS AND METHODS

### PREPARATION OF ORGANOTYPIC CULTURES

All procedures were approved by the animal care committee (Eberhard Karls University, Tübingen, Germany) and were in accordance with German law on animal experimentation. Organotypic slices cultures of rat neocortex were of the “roller-tube” type (Gähwiler, 1981) and prepared as described previously (Antkowiak, 1999). In brief, pups of both sexes aged 2–5 days were deeply anesthetized with isoflurane and decapitated. Following the brain’s removal from the skull coronal slices of 300 μm thickness were cut on a vibratome (Campden Instruments) in ice-cold dissection buffer consisting of 2.0 mM CaCl_2_, 5.0 mM KCl, 0.22 mM KH_2_PO_4_, 0.84 mM Na_2_HPO_4_, 11 mM MgCl_2_, 0.3 mM MgSO_4_, 120 mM NaCl, 27 mM NaHCO_3_, and 60 mM D-glucose. Pieces of neocortex of 2–3 mm length (somatosensory areas according to Paxinos and Watson, 1986) were excised from the slices and fixed on glass coverslips by a plasma clot. The fixed slices were transferred into plastic tubes containing 750 μL of a serum-based nutrition medium and kept in a roller drum at 36°C. One day after preparation, the medium was changed and antimitotics were added; otherwise the nutrition medium was renewed twice a week. After preparation and after each medium renewal the cultures were incubated for 1–2 h in an atmosphere of 5% carbon dioxide in room air, which established of a pH of 7.2–7.4 in the medium. Cultures were used for recordings after 2 weeks *in vitro*.

### ELECTROPHYSIOLOGY

The slice cultures were perfused at a rate of 1 mL/min with artificial cerebrospinal fluid (aCSF) consisting of (in mM): 120 NaCl, 3.3 KCl, 1.13 NaH_2_PO_4_, 26 NaHCO_3_, 1.8 CaCl_2_, 0.2 MgCl_2_, and 11 D-glucose, equilibrated with 95% oxygen/5% CO_2_. We deliberately chose a concentration of magnesium which is lower than *in vivo* (~0.7–1.0 mM) and elevates neuronal excitability. All recordings were performed at 34°C.

Extracellular recordings were performed with glass electrodes filled with aCSF (2–5 MΩ). Usually, pairs of electrodes were inserted in infragranular layers at opposing horizontal positions such that interelectrode distance was 500–1000 μM, about a third of the horizontal extent of the networks, but occasionally one or both electrodes were inserted in supragranular layers in order to maximize the signal to noise ratio. Broadband signals were amplified and bandpass filtered (passband 1–5000 Hz) with an AM-1800 (A-M systems) or Multiclamp 700A (Molecular Devices, Sunnyvale, CA, USA) amplifier. Whole-cell current clamp and voltage clamp recordings were performed in infragranular layers with borosilicate electrodes pulled to a resistance of 2.5–5 MΩ. Intracellular solution for current clamp recordings consisted of (in mM) K-gluconate 135, HEPES 10, EGTA 10, CaCl_2_ 0.5, MgCl_2_ 2.0, Na_2_ATP 3.0, NaGTP 0.3, Na_2_phosphocreatine 10.0, pH 7.3. In a third of the recordings, the fluorescent dye Alexa Fluor 555 (Invitrogen) was included at 50 μM in the solution. All reported membrane potential values were corrected for the calculated liquid junction potential of -17.0 mV. Intracellular solution for voltage clamp recordings contained (in mM) Cs-gluconate 120, HEPES 10, EGTA 10, CaCl_2_ 0.5, MgCl_2_ 2.0, Na_2_ATP 3.0, NaGTP 0.3, Na_2_phosphocreatine 10.0, QX-314 4, pH 7.3. Holding potentials were compensated for the calculated liquid junction potential of -17.6 mV. Access resistances were typically 10–20 MΩ and were compensated 20–40% (voltage clamp). The neurons were recorded at holding potentials of -86 and 0 mV to obtain predominantly glutamatergic and GABAergic current estimates, respectively. Extra- and intracellular signals were digitized at 10 or 20 kHz via a Digidata 1440 interface and pClamp 10 software (Molecular Devices, Sunnyvale, CA, USA). Neuronal activity was usually recorded with two electrodes per culture, in various configurations (both extracellular, both intracellular, or mixed).

For an electrophysiological characterization of neuronal cell types, neurons recorded in current clamp were injected with brief hyperpolarizing current steps of fixed amplitude. During neuronally silent periods, depolarizing current steps of increasing amplitude were injected to elicit APs. Neurons with a pyramidal appearance and/or an AP width of at least 1.5 ms (measured at half-amplitude) and accomodating firing pattern were classified as putative pyramidal cells; non-pyramidal neurons with an AP width of at most 0.7 ms were classified as putative fast-spiking cells; all other neurons were not classified. We did not partition the results according to cell type. Somatic excitability of the neurons was tested with a sinusoidal ramp current injection. The sine wave component, composed of eight full periods, had a frequency of 40 Hz and a peak-to-trough amplitude of one-fifth of the maximum value of the ramp (**Figure 7A**). Current amplitudes were adjusted such that under control conditions and in those sweeps occurring immediately before a burst APs were evoked on the fourth or fifth peak so that drug-induced alterations of excitability in both directions would register. The amount of network activity going on during the stimulus was computed as the difference between the 2.5th and 97.5th percentile of the voltage trace in an interval of 100 ms immediately preceding the stimulated response. Any AP in this interval was removed by substituting the AP waveform (in a 4 ms interval) by a linear interpolation of the membrane potential surrounding the AP.

### DRUGS

NO-711 hydrochloride, SNAP-5114, bicuculline, and muscimol were purchased from Sigma. CGP 55845 hydrochloride was obtained from Tocris. All drugs were applied via the bath solution. On the day of experiments, the drugs were prepared from stock solutions stored at -28°C. For stock solutions, NO-711 hydrochloride, bicuculline, and muscimol were dissolved in water. SNAP-5114 and CGP 55845 were dissolved in dimethylsulfoxide (DMSO). The presence of DMSO at the resulting dilution (maximally 0.1% for SNAP-5114, maximally 0.05% for CGP 55845) had previously not been found to alter neuronal activity in cortical cultures.

### DATA ANALYSIS

#### Extracellular signals

**FIGURE 1:**
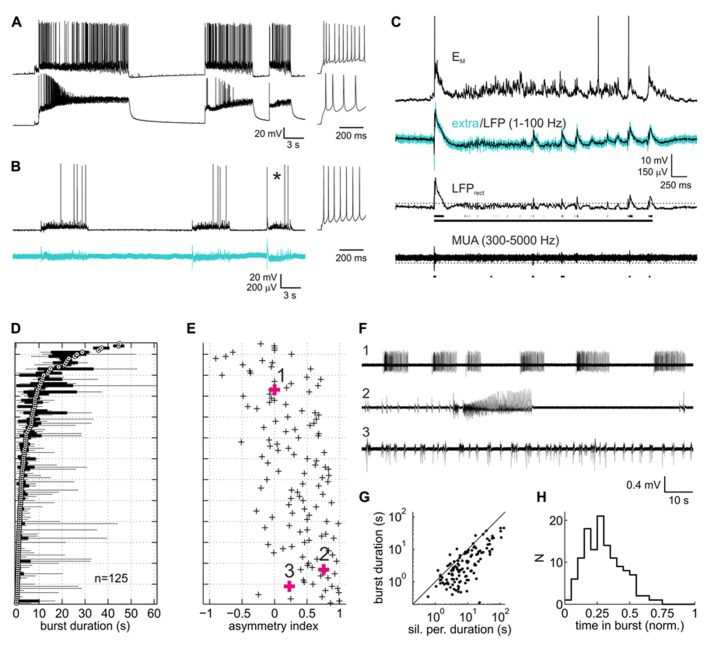
**Spontaneous activity patterns in organotypic slice cultures of rat neocortex.** **(A)** Paired current clamp recording of a putative fast-spiking interneuron (upper trace) and a putative pyramidal neuron (lower trace) which were separated by ~150 μm and not connected to each other. Insets to the right represent spike responses to depolarizing current injections during silent periods. Both neurons were in infragranular layers. **(B)** Example of a paired current clamp (upper trace) and extracellular recording (lower trace, broadband signal, 1–5000 Hz). As in **(A)**, inset depicts the neuron’s spiking response. **(C)** Expanded view of burst marked by asterisk in **(B)**. Upper trace represents the current-clamped neuron’s membrane potential with action potentials clipped at -20 mV. Turquoise and overlaid black trace in the second row from top represent the extracellular broadband trace and the local field potential (LFP) component, respectively. Third trace from top is the rectified LFP trace (LFP_rect_) which was used to detect bursts via a threshold (dotted line). Scattered line fragments below the trace are the portions of LFP_rect_ above threshold. Continuous line represents the burst as defined on the basis of a maximally allowed gap width of 500 ms between the fragments (see Materials and Methods for more detail on burst detection). Bottom trace, highpass filtered extracellular signal containing multiunit activity. Dots below the trace are the action potentials detected via a threshold (dotted line), which in the example shown was at -4.4 standard deviations of the base line noise. **(D)** Box and whisker plot of burst duration. Each sample represents a recording from one culture under control (drug-free) condition (*n* = 125, pooled from all experiments). Data are sorted according to the median (white circle with black central dot). Black bars depict the interquartile range, and black lines extend to the full range of burst durations in the recordings. **(E)** Asymmetry index (ai, see Methods) of burst duration of the corresponding recordings. Positive/negative values indicate the presence of a few disproportionately long/short bursts (relative to the median). **(F)** Representative extracellular recordings (broadband signal) marked by bold symbols and numbers in **(B)**. **(G)** Plot of median burst duration vs. median of silent period duration for each of the recordings shown in **(D)** and **(E)**. Solid line is the identity line. **(H)** Distribution of proportion of time in bursts.

The data recorded from each extracellular electrode were digitally filtered offline. They were split up into a multiunit AP component (highpass, -3 dB corner frequency 300 Hz) and a local field potential (LFP) component (lowpass, -3 dB corner frequency 100 Hz). APs were determined via a simple threshold algorithm. An extracellular AP was defined as a reversible excursion of the highpass filtered signal from baseline beyond a threshold, the first crossing of the threshold defining the spike’s time of occurrence. The (positive or negative) threshold was set manually to at least four standard deviations of the whole data trace above or below the base line. The number of APs divided by recording duration yielded the average multiunit firing rate. LFPs were also fed into a threshold algorithm to determine recurrent phases of network activity, here termed bursts, and phases of neuronal quiescence, termed silent periods. In a first step, the LFP signal was rectified. Next, as in the case of APs, excursions of the rectified LFP beyond a manually set threshold were determined. Series of such excursions were defined as being part of a single burst if the gaps between them did not exceed 400–700 ms, depending on activity patterns (see **Figure 1C** for an example). Single excursions with a duration of less than 20 ms usually represented artifacts and were rejected. Peri-burst time histograms (PETHs) of multiunit APs were constructed by defining the beginning of LFP bursts as time zero and averaging the bin-wise firing rates on the same electrode across all bursts of a recording.

We found that gross patterns of activity recorded on pairs of electrodes were very similar to each other (see Results). Furthermore, upon drug application, burst duration and other LFP-derived parameters covaried strongly among recording sites, as did average firing rates. Therefore, all parameters investigated here were first computed for each electrode and then averaged across electrodes, yielding one sample for each recording. In drug application experiments the data were normalized with respect to the control condition (absence of the tested drug).

Effects of the drugs on activity *levels* were assessed on the basis of two parameters, the average firing rate as explained above and the proportion of time the networks spent in bursts (the sum of all burst durations divided by recording duration). In the case of NO-711, a Hill fit to the concentration–response relationship of either parameter yielded the half-effect inhibitory concentrations (IC_50_); as SNAP5114 and muscimol depressed firing in a qualitatively different manner, data from these experiments were not fit. Activity *patterns* were described in terms of burst duration, silent period duration, and PETHs. A median-based method was used to quantify the irregularity of burst durations. The asymmetry index (ai) was computed as

$$ai\text{ =}\frac{\left(95th\,\;percentile\,\;-\;median)\;-\;(5th\,\;percentile\,\;-\;median\right)}{95th\,\;percentile\,\;-\;5th\,\;percentile}$$

This measure compares extremes of burst durations above and below the median. It ranges between -1 (the shortest bursts are far more distant from the median than are the longest) and 1 (the inverse); values close to zero indicate a symmetric distribution of burst durations relative to the median.

#### Voltage clamp recordings

Phasic currents were analyzed as described in detail in the main text (**Figure 5**). Drug-induced changes of tonic currents were assessed as changes of the holding current in the silent periods between bursts. The current traces were subdivided into segments of 200 ms; the peaks of the amplitude histograms of these were averaged per condition to yield the holding current.

#### Current clamp recordings

Spontaneous and stimulated APs were detected via a threshold algorithm. Intracellular membrane potential traces very well reflected the network activity visible in extracellular signals and were therefore also used to determine bursts and silent periods; these were pooled with the extracellular data. Single exponentials were fit to the membrane potential deflections stemming from hyperpolarizing current injections; from these membrane decay time constant and resistance were computed. Neuronal excitability was assessed as the number of APs elicited by the sinusoidal ramp current injection described above.

### STATISTICAL ANALYSIS

Statistical analysis in this study is based on measures of effect size (MES) and 95% confidence intervals thereof (CI95; Kline, 2004; Nakagawa and Cuthill, 2007; Hentschke and Stüttgen, 2011). Unless otherwise mentioned, CI95 are given in square brackets after the MES in question and *p* values of corresponding hypothesis tests, if amenable, are given as complementary information.

In one-way and two-way factorial analyses, eta squared (η^2^) and partial eta squared ($\eta_{p}^{2}$) were computed to estimate the amount of variance in the data explained by the independent variable(s) (drug concentrations) and their interaction. Like other squared correlation-type MES, η^2^ and $\eta_{p}^{2}$ range from zero (no effect) to one; the closer the value is to one, the stronger the effect.

For comparisons of two groups of data, we used the following standardized mean differences: md/sd (mean difference divided by the standard deviation of the difference score; paired data) and g_ψ_ (weighted mean differences of groups divided by the population standard deviation; paired data within one-way factorial analyses). For a comparison of one data group against a fixed control value of 1 we used *g*_1_ (difference of the group mean to the fixed value divided by standard deviation of the group). These MES can attain any value; the further a concrete value deviates from the “null effect” value of zero, the stronger the effect.

As a multivariate estimate of the difference in activity patterns between two drug conditions, we used Mahalanobis distance (D). D is a measure of the distance between the means of the two compared populations in multidimensional space, expressed in multiples of the standard deviations pooled across both populations. It can thus be regarded as a multivariate extension of standardized mean differences (Del Giudice et al., 2012).

Two parameters investigated here, multiunit firing rate in peri-event histograms and current slopes in voltage-clamp experiments, showed skewed distributions or largely different variances between the groups to be compared. Accordingly, these data were dealt with via a non-parametric statistic, area under the receiver-operating curve (AUROC). AUROC is closely related to the Mann–Whitney U statistic and ranges from zero to one; it can be used as a measure of the difference between two populations (Bamber, 1975; Brown and Davies, 2006): a value of 0.5 indicates complete overlap of (i.e., no difference between) the two compared populations; the further its value deviates from this “null effect” value, the stronger the effect. CI95 of this measure as well as of D were computed via bootstrapping, whereas in all other cases analytical CI95 were computed. Formulae for all MES except D are given in (Kline, 2004) and the documentation of the Measures of Effect Size Toolbox (Hentschke and Stüttgen, 2011). The Measures of Effect Size Toolbox and in-house software written in Matlab was used for all analyses.

## RESULTS

Slice cultures of rat neocortex showed a high degree of spontaneous neuronal network activity. Intracellularly, activity was visible as phases of intense synaptic input which depolarized the neurons and triggered AP activity (**Figures 1A,B**). These phases of activity, here termed “bursts,” consisted of an initial strong depolarizing bout, often triggering high-frequency AP firing. This was usually followed by a rapid drop of the membrane potential and a phase of less intense firing and decelerating broadband (2–40 Hz) oscillatory reverberations. The oscillatory reverberations could show a slow hyper- or depolarizing trend toward the end of the burst. In extracellular recordings, bursts appeared as an initial strong deflection of the field potential and high-frequency multiunit AP firing, followed by reverberatory patterns paralleling those seen intracellularly (**Figures 1B,C**). Gross activity patterns were always very similar on pairs of electrodes. Bursts overlapped in time by 88.5% (median of all paired recordings under control; interquartile range 77.7–94.1%), suggesting that the bursts pervaded large portions of the cultures. Bursts were separated by phases of relative neuronal quiescence (“silent periods”) with low levels of subthreshold synaptic input. AP firing in silent phases was very low, accounting for only 1.8% of all spikes (median; interquartile range 0.4–4.3%).

Burst duration varied considerably between individual cultures, as has been noted before for both organotypic and dissociated cortical cultures (Wagenaar et al., 2006; Johnson and Buonomano, 2007; Czarnecki et al., 2012). The reverberatory phase accounted for most of the variability of burst duration both within and between individual networks (**Figure 1D**). Networks with a short median burst duration showed a clear tendency to produce occasional long bursts, as indicated in the positive bias of the ai (see Materials and Methods) of burst duration for these networks (**Figure 1E**). In some of these networks a series of brief bursts of increasing intensity and duration culminated in an extremely long burst, followed by a prolonged period of neuronal silence, after which the sequence restarted (**Figure 1F**, second trace). Overall, silent periods lasted longer than bursts, and their median duration correlated well with the median duration of bursts (**Figure 1G**, *r* = 0.84 [0.78 0.89]). Consequently, the proportion of time the networks spent in bursts was comparatively homogeneous, centered around a median of 0.28 (**Figure 1H**; interquartile range 0.18–0.38). Average multiunit firing rates were variable between cultures (median 5.1 Hz, interquartile range 1.4–8.6 Hz), in all likelihood due to the variable number of neurons recorded from per electrode. Overall, activity patterns combined features typical of networks hyperexcited by nominally zero extracellular magnesium (Gutnick et al., 1989; Kawaguchi, 2001) and features reported in previous studies on rat neocortical slice cultures in less activity-fostering conditions (Plenz and Kitai, 1996; Klostermann and Wahle, 1999; Hentschke et al., 2005; Johnson and Buonomano, 2007; Czarnecki et al., 2012).

### GAT-1 vs. GAT-2/3

**FIGURE 2:**
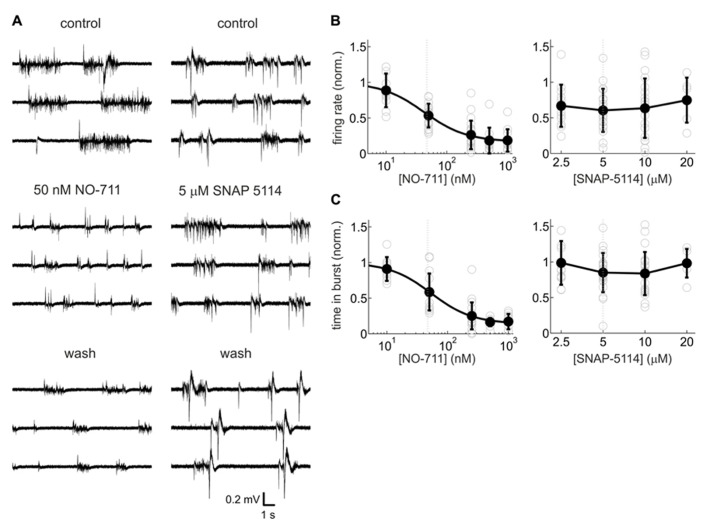
**Activity-depressant effects of the GAT-1-selective blocker NO-711 and the GAT-2/3 preferring antagonist SNAP-5114.** **(A)** Example extracellular recordings (broadband signal) with both drugs at concentrations close to the IC_50_ of their most sensitive molecular targets. 50 nm NO-711 diminished spontaneous activity strongly, whereas 5 μM SNAP-5114 had a weaker effect. The effects of NO-711 were only partly reversible upon washout (Gonzalez-Burgos et al., 2009). **(B)** Concentration–response curves of NO-711 (left) and SNAP-5114 (right) for multiunit action potential rates. All data are normalized to the control condition (absence of drug). Open gray circles are data from individual experiments, filled black circles and error bars represent means and sd, respectively. Smooth black line connecting the means represents a Hill fit to the NO-711 data. Gray dashed lines represent IC_50_ values. **(C)** Concentration–response curves of NO-711 (left) and SNAP-5114 (right) for the proportion of time spent in bursts. All conventions as in **(B)**.

In previous studies in neocortex, blockade of GAT-1 was found to substantially modulate GABAergic inhibition, whereas blockade of GAT-2/3 produced mixed results (Keros and Hablitz, 2005; Kinney, 2005; Bragina et al., 2008; Gonzalez-Burgos et al., 2009). In order to assess the roles of the different GATs in shaping spontaneous activity, we compared neuronal activity changes caused by the GABA uptake inhibitors NO-711 (GAT-1 antagonist) and SNAP-5114 (GAT-2/3 antagonist). **Figure 2** shows that the selective GAT-1 blocker NO-711 inhibited activity in a concentration-dependent fashion. Average firing rates, normalized to control, were well fit by a Hill curve (adjusted *R*^2^ = 0.63) and the IC_50_ of 42 nM was close to the IC_50_ of 47 nM for inhibition of GAT-1 in rat synaptosomes (Suzdak et al., 1992). At 1000 nM NO-711, neuronal activity was depressed by over 80% (**Figure 2B**, left). Very similar results were obtained by assessing the proportion of time spent in bursts (**Figure 2C**, left; IC_50_ = 53 nM, adjusted *R*^2^ = 0.67). By contrast, the GAT-2/3 preferring antagonist SNAP-5114 depressed AP activity on average by about 30%, with no consistent dependence on concentration (**Figure 2B**, right). Time spent in bursts did not appear to change with drug treatment (**Figure 2C**, right). For a statistical comparison, we expressed the concentrations of both drugs in terms of approximate multiples of IC_50_ of GABA uptake at their most sensitive target and subjected data from roughly matching concentrations to a two-way interaction analysis with the GAT targeted as the first factor and drug concentration as the second factor. SNAP-5114 has an IC_50_ of GABA uptake of 5 μM at GAT-3 (20 μM at GAT-2; Borden et al., 1994); hence, the following data were compared: NO-711, 50 and 250 nM (1.06 and 5.32 in IC_50_ multiples) with SNAP-5114, 5 and 20 μM (1.00 and 4.00 in IC_50_ multiples; Suzdak et al., 1992). The results confirmed that GAT-1 inhibition had more consistent effects than GAT-2/3 inhibition: partial eta squared ($\eta_{p}^{2}$) for the interaction [drug type × normalized concentration] was 0.132 [0.016 0.290] (*p* = 0.0034) for firing rates and 0.141 [0.021 0.296] (*p* = 0.0019) for proportion of time spent in bursts. Hence, all further experiments were conducted with NO-711.

**FIGURE 3:**
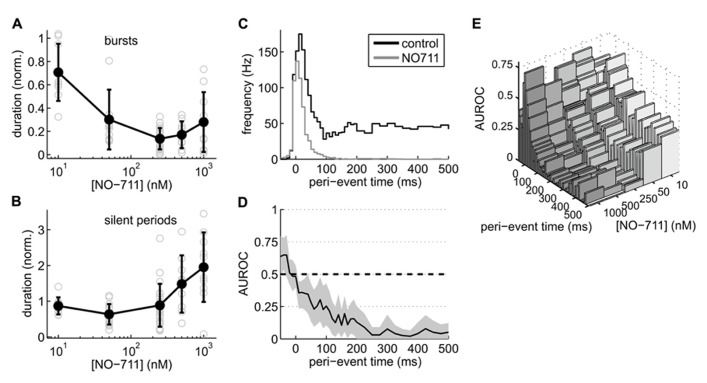
**Effects of NO-711 on activity patterns.** **(A)** Concentration–response plot of burst duration normalized to control (means ± sd; gray open symbols are data from individual experiments). **(B)** Concentration–response plot of silent periods [same conventions as in **(A)**]. Note different scale of ordinate. **(C)** Peri-burst time histograms of multiunit firing activity during control and 250 nM NO-711 (*n* = 16, paired data, median of all experiments). *t* = 0 is the beginning of bursts as detected in the field potential. Bin widths were 10 ms up to 200 ms post-event and 25 ms further into the bursts. **(D)** AUROC (black line) and bootstrapped 95% confidence intervals (gray area) for the bin-by-bin comparison of the PETHs in **(C)**. **(E)** Same as **(D)**, but for all concentrations of NO-711 tested. Confidence intervals were omitted for clarity.

Closer inspection of activity patterns revealed that bursts were drastically shortened at all concentrations of NO-711, whereas the silent periods between bursts showed a biphasic concentration dependence (**Figures 3A,B**): concentrations up to 250 nM NO-711 on average shortened silent periods, resulting in an increased burst frequency as visible in the example recording in **Figure 2A**, whereas higher concentrations prolonged silent periods, possibly due to tonic currents which may emerge at these concentrations (see Discussion). Moreover, AP firing within the bursts was depressed in a progressive fashion (**Figure 3C**). Under control, median firing frequency peaked 20 ms after burst begin and settled to a constant level after about 150 ms. At 250 nM NO-711, firing was mostly restricted to an initial bout of less than 100 ms duration. The more in-burst time progressed, the stronger the depression (**Figure 3D**). This effect depended on the concentration of NO-711 (**Figure 3E**).

### GABA_A_ vs. GABA_B_ RECEPTORS

**FIGURE 4:**
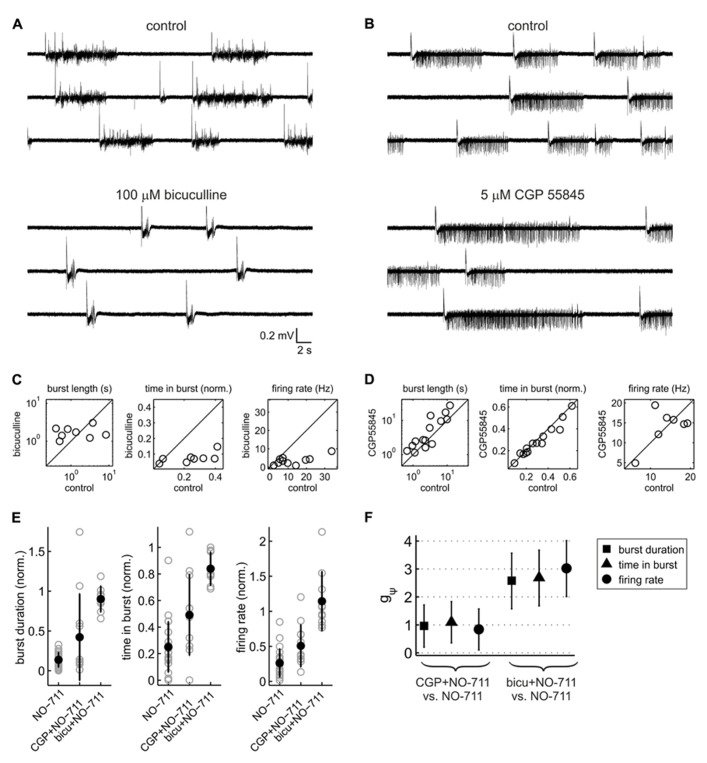
**Contribution of GABA_A_ and GABA_B_ receptors to activity patterns and to the depressant effects of NO-711.** **(A)** Exemplary extracellular recording of a culture exposed to the GABA_A_ receptor antagonist bicuculline (broadband signal). Note the drug-induced stereotyped appearance of the network bursts. **(B)** Exemplary extracellular recording with the GABA_B_ receptor blocker CGP55845 (5 μM).** (C)** Effect of bicuculline (100 μM) on median burst duration (left), proportion of time spent in bursts (center), and average firing rate (right). Bicuculline appeared to “clamp” burst duration at 1.8 ± 0.7 s (mean ± sd), on average causing a prolongation with wide margins of uncertainty (*g*_1_(1) = 0.52 [-0.24 1.25], *p* = 0.18, *n* = 8); it strongly reduced both the proportion of time spent in bursts (*g*_1_(1) = -1.58 [-2.62 -0.49], *p* = 0.003, *n* = 8) and average firing rates (*g*_1_(1) = -2.60 [-3.90 -1.25], *p* = 1.8 * 10^-5^, *n* = 10). **(D)** Effect of CGP55845 (5 or 10 μM) on the same parameters as shown in **(C)**. CGP55845 increased burst duration on average by 68% (*g*_1_(1) = 0.76 [0.15 1.35], *p* = 0.014, *n* = 14), whereas the proportion of time spent in bursts showed a weak opposite effect (*g*_1_(1) = -0.45 [-0.99 0.11], *p* = 0.14, *n* = 14). There was no evidence for an effect on average firing rates (*g*_1_(1) = 0.11 [-0.63 0.85], *p* = 0.77, *n* = 7). **(E)** Comparison of the effects of 250 nM NO-711 alone and with prior application of CGP 55845 and bicuculline: median burst duration (left), proportion of time spent in bursts (center) and average firing rate (right). All data were normalized to the respective control (absence of NO-711). Full symbols and error bars are means ± sd; gray symbols are data from individual experiments. **(F)** Statistical analysis of the data shown in **(E)**. The plot depicts, for each of the three parameters shown in **(E)**, standardized mean differences (termed *g*_ψ_) between the conditions [NO-711] and [GABA receptor antagonist and NO-711]. Error bars are 95% confidence intervals.

Thus far, the data demonstrate that impairment of GAT-1 inhibits cortical spontaneous activity, possibly by enhancing spillover of GABA from the synaptic release sites to peri- and extrasynaptic GABA receptors. In order to assess the contribution of GABA_A_ versus GABA_B_ receptors to the inhibitory effects of NO-711, we performed experiments in which blockers of either receptor type were applied prior to NO-711. GABA_A_ receptor blockade by 100 μM bicuculline transformed activity into a regular pattern of stereotyped bursts characterized by intense firing activity, strong oscillatory (~10 Hz) components and prolonged silent periods between bursts, typical of disinhibited cortical networks (Gutnick et al., 1989; Castro-Alamancos et al., 2007; Sanchez-Vives et al., 2010; **Figures 4A,C**). By contrast, GABA_B_ receptor blockade by CGP 55845 (5 or 10 μM) altered activity in a moderate and selective manner, primarily by prolonging bursts (**Figures 4B,D**). **Figures 4E,F** illustrate that both GABA_A_ and GABA_B_ receptor blockade curtailed the inhibitory effect of 250 nM NO-711 [one-way factorial analysis with pretreatment (none, GABA_A_ receptor antagonism, GABA_B_ receptor antagonism) as the independent factor; median burst duration: η^2^ = 0.51 [0.26 0.64], *p* = 1.2 * 10^-6^, df = 40; proportion of time spent in bursts: η^2^ = 0.53 [0.28 0.65], *p* = 3.3 * 10^-^^7^, df = 42; average firing rate:η^2^ = 0.61 [0.38 0.72], *p* = 7.2 * 10^-9^, df = 42]. As expected, when GABA_A_ receptors were blocked, the inhibitory effect of NO-711 was to a large degree diminished, suggesting that it was mostly, but not exclusively, mediated by GABA_A_ receptors. Results from complementary experiments (prior blockade of GABA_B_ receptors) confirmed this notion, as is visible in a statistical comparison of the effects of NO-711 in both scenarios versus the effects of NO-711 *per se* (**Figure 4F**). Taken together, the results confirmed that GABA_A_ receptors make the strongest contribution to inhibition and that GABA_B_ receptors shape spontaneous activity by curbing the duration of network bursts (Mann et al., 2009). They also suggest that like GABA_A_ receptors, GABA_B_ receptors experience stronger exposure to GABA during GAT-1 impairment (Thompson and Gähwiler, 1992).

### MECHANISM OF NETWORK INHIBITION BY GAT-1 ANTAGONISM

**FIGURE 5:**
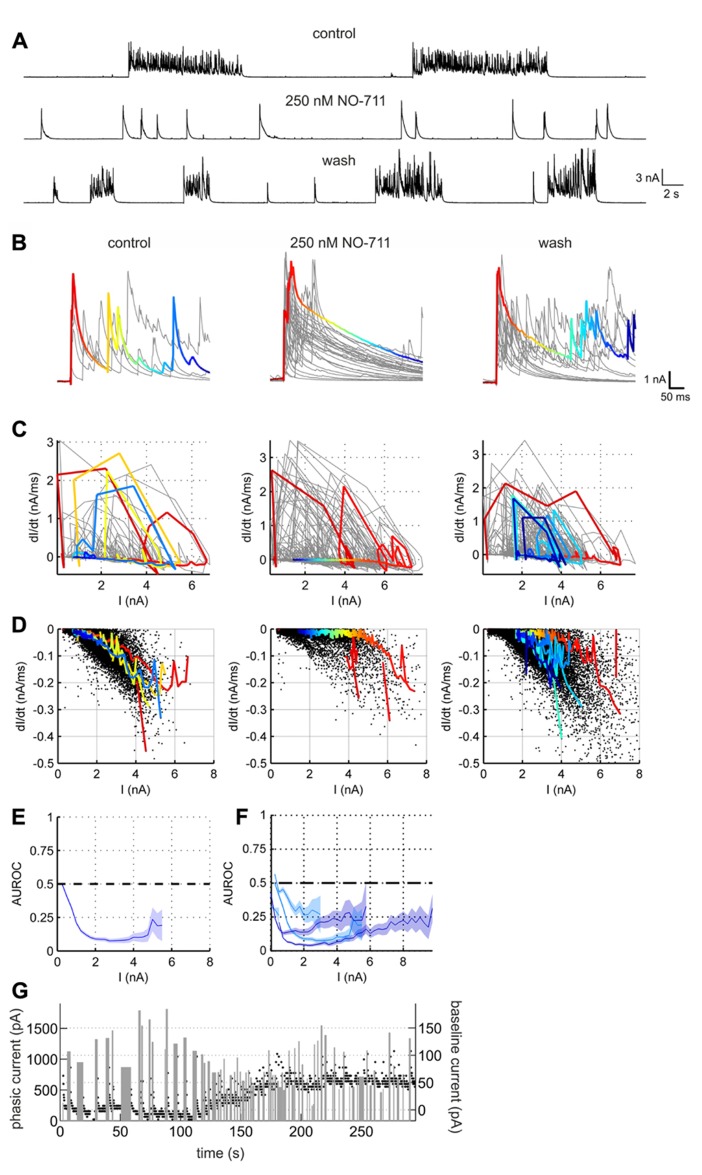
**GABAergic currents altered by GAT-1 inhibition.** **(A)** Example recording of a putative pyramidal neuron held in voltage clamp at 0 mV, revealing large outward currents which reflect mostly chloride currents through GABA_A_ receptors. The effects of NO-711 were only partly reversible after washout. **(B)** Excerpts of outward currents triggered to the onset of the bursts. In all three conditions, one representative burst is depicted in color, the hue corresponding to time. **(C)** Plots of current slope vs. current amplitude for the excerpts shown in **(B)** (same column order). Colored trajectories correspond to the colored excerpts in **(B)**. Jagged appearance of positive slopes (rise phase) is due to downsampling of data to 1000 Hz (after lowpass filtering at 500 Hz). **(D)** Plots of current slope vs. current amplitude for all excerpts at full length in the recording shown in **(A)**, restricted to the decay phases (negative slopes). Each dot represents one linear slope. Colored trajectories are the same as in **(C)**, except that segments with a positive slope were omitted. Note the upward shift of points toward less negative values in the middle graph, corresponding to a flattening of the decay currents. **(E)** Comparison of the current decay slopes in the control condition with those in the presence of NO-711. The blue line depicts the AUROC values stemming from an amplitude-matched comparison of the current slope values shown in **(D)** (bin size was 0.25 nA). Shaded area corresponds to bin-wise 95% confidence intervals. The “zero effect” value of AUROC at 0.5 is marked with a dashed line. **(F)** Summary statistics for five tested neurons (cyan, 250 nM NO-711, blue, 1000 nM NO-711). **(G)** Development of tonic and phasic outward currents during wash-in of 1000 nM NO-711. Black dots are estimates of the holding current averaged in 200 ms-intervals; width and height of gray bars represent duration and amplitude averaged over duration, respectively, of phasic currents. Note the different amplitude scales.

Next, in order to assess alterations of GABAergic inhibition due to GAT-1 impairment we performed voltage clamp recordings in the presence of full network activity. **Figure 5** depicts the NO-711-induced transformation of currents in a putative pyramidal neuron, voltage-clamped at a holding potential of 0 mV to reveal inhibitory GABAergic postsynaptic currents. Under the drug-free condition the currents were grouped in clearly discernible bursts of numerous overlapping transients with a fast rise time and a slower decay, reflecting compound IPSCs (cIPSCs). In the presence of 250 nM NO-711, individual bursts were mostly reduced to one large, fast-rising transient with a decay which was noticeably slower than the decay of the multiple individual transients in the bursts under control (**Figure 5B**). As the individual transients overlapped substantially we could not extract decay time constants from them. Instead, in order to quantify the drug-induced changes in current decay we compared the piecewise linear slopes of the currents under control and NO-711. This was done in an amplitude-matched way: in the phase plane defined by current amplitude (I) and current slope (dI/dt; **Figures 5C,D**), all points with a negative slope (depicted in **Figure 5D**) were binned according to amplitude (bin width 0.25 nA). The rationale for this approach was that the slope of a single exponential is directly proportional to its amplitude, and that prolongation of GABAergic currents due to GAT-1 inhibition was expected to depend on the amount of GABA released, reflected in current amplitude. Then, for each neuron individually, the slopes were compared bin-wise between the control condition and NO-711 via AUROC (**Figures 5E,F**). It is quite evident that amplitude-matched slopes during the decay phase of the cIPSCs were consistently shallower in the drug condition for all five neurons tested (**Figure 5F**). Thus, the experiments were in agreement with the hypothesis of an enhanced activity-dependent spillover of GABA due to inhibition of GAT-1. Effects of GAT-1 inhibition on tonic currents were inconclusive: while in one neuron exposed to 1000 nM NO-711 base line currents were essentially unaltered (increase by 8 pA), they increased in the other four neurons by between 45 and 255 pA (an example is shown in **Figure 5G**).

**FIGURE 6:**
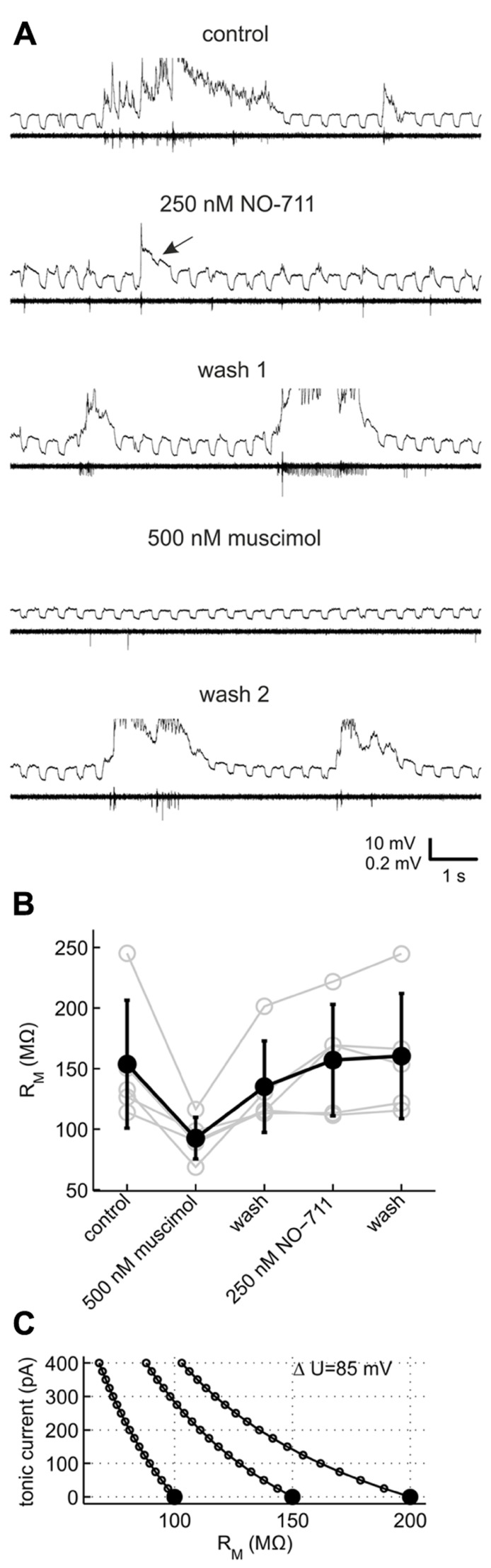
**Effects of GAT-1 inhibition and tonic activation of GABA_A_ receptors on neuronal membrane resistance.** **(A)** Example recording of a putative pyramidal neuron (top trace within each pair of traces, *R* = 150 MΩ) and multiunit action potential activity on a nearby extracellular electrode (bottom trace) during control and after sequential application of NO-711 and muscimol. The neuron was repetitively injected with a 40 pA rectangular hyperpolarizing current throughout the length of the data excerpts shown. The current traces are clipped to reveal detail of the responses to current injection. Arrow in the second pair of traces from top points to a membrane voltage response which is strongly reduced in the wake of a network burst in 250 nM NO-711. Note the nearly complete absence of spontaneous activity with 500 nM muscimol which recovers after the second washout. **(B)** Resting membrane resistance of five neurons exposed to both muscimol and NO-711. **(C)** Simplified theoretical relationship between an observed change of resting membrane resistance (abscissa) and the underlying tonic conductance, expressed in terms of the tonic current at an assumed ionic driving force of 85 mV. Curves are shown for three neurons with different initial resistances (full symbols).

For methodological reasons we did not attempt to quantify phasic or tonic currents more precisely (see Discussion), but instead performed current clamp recordings to investigate which mode of inhibition was preferentially fostered by NO-711. We monitored neuronal membrane resistance and excitability, which should be differentially affected by changes in tonic and phasic inhibition. For example, in a typical neuron with a membrane resistance of 150 MΩ, the conductance underlying a tonic GABAergic current of 100 pA in our experimental conditions should decrease the neuron’s resistance at rest by 15% (**Figure 6C**). By contrast, increases in phasic inhibition should register as decreases of neuronal resistance and excitability time-locked to the bursts. To validate the general approach, the effects of NO-711 were compared with those of muscimol, a GABA mimetic which is not transported by GAT-1 and should therefore induce an activity-independent tonic current. We chose a concentration of 500 nM muscimol, which was comparable to 250 nM NO-711 in terms of depression of firing activity (cf. **Figures 2B** and 8A).

**Figure 6** shows an exemplary recording of a pyramidal neuron which was repetitively injected with negative current steps to probe membrane resistance. During network bursts under control conditions, membrane responses were obscured by strong fluctuations of the membrane voltage. After burst offset, the voltage responses were smaller than pre-burst values, recovering gradually. In the presence of 250 nM NO-711, the voltage responses were obscured by the frequent, brief network bursts, and strongly reduced in amplitude in the wake of occasional longer bursts (**Figure 6A**, arrow), but in other phases did not appear different from pre-burst responses under control. 500 nM muscimol, by contrast, reversibly suppressed network activity and decreased the voltage responses strongly, independent of the occurrence of bursts. As the distortion of the voltage responses during network activity under any condition did not allow for a reliable estimation of membrane resistance, we chose responses to current injections preceding the onset of bursts by up to 4 s for a comparison of the different drug conditions. In five neurons which could be held over the course of two drug applications and washout phases, muscimol consistently and strongly diminished membrane resistance compared to control (**Figure 6B**; comparison of muscimol condition to a weighted average of control and both washout values, dependent data: *g*_ψ_ = 1.32 [0.78 1.87], *p* = 9.19 * 10^-^^5^). By contrast, NO-711 had a small effect on pre-burst membrane resistance (*g*_ψ_ = -0.17 [-0.71 0.37], *p* = 0.51). In these experiments, NO-711 was applied as the second drug except in the case depicted in **Figure 6A**, so that an incomplete washout of muscimol may have masked effects of NO-711. However, 10 additional recordings with 250 nM NO-711 as the sole application confirmed that the drug affected pre-burst membrane resistance only weakly, on average diminishing it by less than 10% (control, 100 ± 38 Mømega (sd); NO-711, 92 ± 43 Mømega; *g*_ψ_ = 0.20 [-0.18 0.58], *p* = 0.26, *n* = 10, data not shown).

**FIGURE 7:**
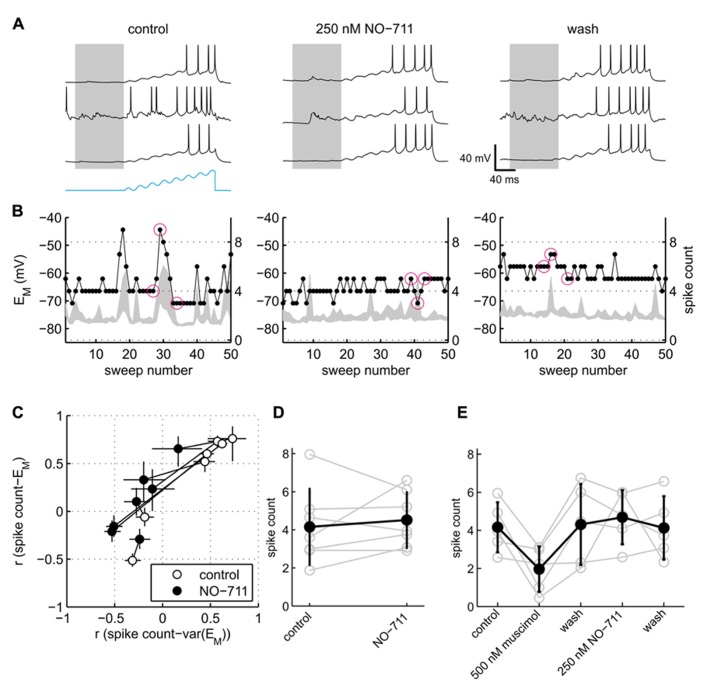
**Effects of GAT-1 inhibition and tonic activation of GABA_A_ receptors on somatic excitability.** **(A)** Exemplary neuronal membrane voltage traces recorded during injection of a sinusoidal ramp current during control (left), 250 nM NO-711 (center), and after washout of the drug (right). The amplitude of the current waveform (blue trace at bottom in left block of traces) was adjusted for each neuron individually such that during control conditions action potentials were elicited starting at about the fifth of the eight current peaks. Membrane potential traces are clipped at 0 mV to enhance visibility of low-amplitude potential fluctuations. Gray patches delineate the pre-stimulus window of [-100 0] ms in which the median and variability of E_M_ were computed. **(B)** For the same experiments as shown in **(A)**, the plots depict the 2.5–97.5 interpercentile range of E_M_ in the pre-stimulus window (gray shades) and the spike count during the current stimulus (dots connected by lines) versus sweep number. Inter-sweep interval was 620 ms. The sweeps marked by magenta circles are depicted in **(A)** (left to right corresponding top to bottom). **(C)** Scatter plot of correlation coefficients *r*_var(EM)_ and *r*_EM_ (see main text) for control and NO-711 conditions. Each pair of circles connected by a dotted line corresponds to one cell. Error bars are 95% confidence intervals. Note that the width of the error bars depends crucially on the number of injected current sweeps, which varied between the neurons and between drug conditions. **(D)** Spike counts, averaged over all sweeps during control and 250 nM NO-711, for the same cells as depicted in **(C)** (means ± sd). Each pair of connected gray open circles represents one cell. **(E)** Spike counts from five additional neurons sequentially exposed to muscimol and NO-711 (same as depicted in **Figure 6B**). Same conventions as in **(D)**.

Somatic excitability of the neurons was tested via repeated sinusoidal ramp current injection, and quantified in terms of the number of APs evoked (**Figure 7**). Visual inspection of the data suggested that responses to current injection depended on spontaneous activity. During control conditions, the exemplary neuron shown in **Figure 6A** emitted more APs during spontaneous network bursts and depolarized E_M_ than during quiescence (**Figure 7A**, left). By contrast, in the presence of NO-711, network activity during or immediately preceding current stimulation had rather the inverse effect, impeding APs. To quantify these observations, we computed the correlation between the median pre-stimulus membrane potential and the stimulated spike count (termed *r*_EM_) as well as the correlation between the intensity of pre-stimulus network activity as expressed in the 2.5–97.5 interquartile range of E_M_ and the stimulated spike count (termed *r*_var(EM__)_). The rationale was that both the absolute value of E_M_ and the fluctuations of E_M_ are hallmarks of ongoing activity and determinants of somatic excitability (Paré et al., 1998). A scatter plot of the correlation coefficients (**Figure 7C**) revealed that somatic excitability of five of seven neurons indeed correlated positively with both membrane potential and ongoing network activity. In the presence of 250 nM NO-711, both correlations dropped notably, with *r*_var(EM__)_ even shifting to negative values in four neurons, indicating that previously excitatory ongoing network activity now had a damping influence on somatic excitability. In two neurons, *r*_var(EM)_ and *r*_EM_ were close to or below zero during control and barely changed in the presence of NO-711, suggesting that these neurons were already under strong inhibitory control in the absence of drug, and therefore little affected by increased inhibition.

Excitability of the neurons averaged across all repetitive current injections, irrespective of their timing relative to ongoing activity, was not affected by NO-711 (**Figures 7D,E**). This finding parallels the observation of a barely affected membrane resistance (**Figure 6**); we attribute both to a failure of submicromolar concentrations of NO-711 to induce substantial tonic currents, among other factors (see Discussion). By contrast, muscimol depressed somatic excitability by almost half (**Figure 7E**). Thus, the current clamp recordings pointed to an intensification of phasic inhibition, but not of tonic inhibition, in the presence of 250 nM NO-711.

**FIGURE 8:**
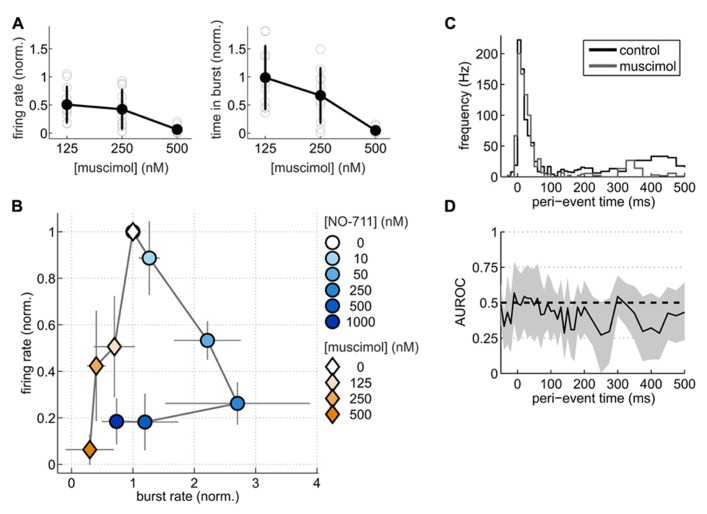
**Spontaneous activity patterns induced by NO-711 and the GABA-mimetic muscimol.** **(A)** Concentration–response relationships of muscimol for average firing rate (left) and proportion of time spent in bursts (right). **(B)** Plot of average firing rate versus burst rate for NO-711 data and muscimol data. The data were normalized to the respective control conditions. Symbols and lines correspond to means and standard deviations, respectively. **(C)** Peri-burst time histograms (PETHs) of median multiunit firing activity during control and 250 nM muscimol (*n* = 9, paired data). **(D)**, AUROC and bootstrapped 95% confidence intervals (shown only for 250 nM) for the bin-by-bin comparison of PETHs during control and drug application. Same conventions apply as in **Figures 3C,D**.

To substantiate this point from another angle, we compared changes of spontaneous activity patterns induced by NO-711 with those induced by muscimol at roughly equipotent concentrations with regard to AP depression. We hypothesized that if NO-711 predominantly enhanced phasic inhibition, it should modulate activity differently from muscimol. Like NO-711, muscimol decreased average firing rates in a concentration-dependent manner (**Figure 8A**). However, the firing patterns imposed by both substances on the networks were vastly different. While NO-711 up to 250 nM shortened both bursts and silent periods (**Figures 3A,B**), resulting in an increased burst frequency, muscimol had the opposite effect (**Figure 8B**). Moreover, while average firing rate and proportion of time spent in bursts showed a very similar dependence on concentration of NO-711 (**Figures 2B,C**), both parameters’ concentration dependence diverged with muscimol (**Figure 8A**). To quantify these differences, we computed the Mahalanobis distance (D) between NO-711 and muscimol data populations in the three-dimensional space defined by burst frequency, proportion of time spent in bursts and average firing rates (all normalized to control). At 50 nM NO-711 (*n* = 15) and 125 nM muscimol (*n* = 10), both of which depressed firing rates by about 50%, activity patterns diverged, as expressed in a Mahalanobis distance of 2.50 [1.90 4.34] between the two populations. Comparing 250 nM NO-711 (*n* = 17) to 250 nM muscimol (*n* = 10) yielded very similar results (*D* = 2.27 [1.63 3.91], *n* = 17 and 10, respectively). Furthermore, firing within bursts was clearly depressed in a progressive way by NO-711 (**Figures 3C–E**), but less so by muscimol (**Figures 8C,D**). Thus, at concentrations depressing AP firing by 50–70%, NO-711 and muscimol imposed largely different firing patterns on the networks.

## DISCUSSION

Impairment of GAT-1 altered neocortical spontaneous activity in drastic ways. Firing rates were depressed and network bursts curtailed at nanomolar concentrations of NO-711: the IC_50_ of firing rates was 42 nM, very close to the IC_50_ of GABA uptake for human GAT-1 (Borden, 1996) and rat GAT-1 as determined in synaptosomes (Suzdak et al., 1992). By comparison, impairing GAT-2/3 was less effective. These findings are in good agreement with previous studies in neocortex reporting strong amplification of stimulated GABAergic inhibition by NO-711 but comparatively moderate effects of SNAP5114 (Keros and Hablitz, 2005; Kinney, 2005). Hence, we focused on the effects of NO-711.

### MECHANISMS OF CORTICAL NETWORK INHIBITION BY GAT-1 ANTAGONISM

The lowest concentrations of NO-711 leading to notable changes in cortical activity patterns in our experiments are minute, at least two orders of magnitude lower than those usually employed for inducing tonic currents (Semyanov et al., 2003; Keros and Hablitz, 2005; Glykys and Mody, 2007b; Gonzalez-Burgos et al., 2009). A discussion of this surprising finding needs to take into account that the effects depend critically not only on the degree of GAT-1 impairment, but also on the amount of GABA released: while the few GABA molecules giving rise to miniature IPSCs simply diffuse away from receptors independent of the presence of GATs, the large amounts of transmitter emitted during synchronous and repetitive activation of numerous GABAergic synapses require GAT-1 for efficient clearance and synapse independence (Overstreet and Westbrook, 2003; Keros and Hablitz, 2005; Gonzalez-Burgos et al., 2009). Under conditions of massive release, the most plausible consequences of mildly impeding GABA uptake by GAT-1 are longer GABA pulses in the synaptic cleft, and enhanced spillover of GABA out of the synaptic cleft. Longer GABA pulses in the synaptic cleft likely recruit additional synaptic GABA_A_ receptors, a proportion of which is not activated or saturated under normal conditions (Hajos et al., 2000; Gonzalez-Burgos et al., 2009; Barberis et al., 2011; Petrini et al., 2011). Enhanced spillover should in addition accentuate the impact of peri- and extrasynaptic receptors as well as of unoccupied synaptic receptors at inactive release sites (Barbour, 2001; Overstreet and Westbrook, 2003). Provided some residual GAT activity is still present and the resulting prolonged exposure of the receptors to GABA is phasic in nature, such that it does not lead to substantial desensitization of the receptors (Overstreet et al., 2000; Keros and Hablitz, 2005), an enhanced or prolonged phasic inhibition should ensue.

A number of our findings argue in favor of this mechanism as the primary cause of depression of network activity observed here. First, inhibitory currents during network activity consisted of numerous overlapping cIPSCs reaching peak amplitudes comparable to those seen in electrically stimulated acute slices, indicative of a repetitive and synchronous release of large quantities of GABA. As would be expected from an impairment of GABA transport close to the release sites, 250 nM NO-711 prolonged the decay phase of the IPSCs. However, two methodological caveats of our experiments must be mentioned: by design, during the network bursts inhibitory as well as excitatory conductances were active, likely aggravating space clamp errors which are inevitable in neurons with extensive dendritic arbors voltage-clamped at the soma (Bar-Yehuda and Korngreen, 2008; Williams and Mitchell, 2008). Moreover, in order to discriminate glutamatergic and GABAergic currents, the neurons had to be held at largely different potentials (-86 and 0 mV). At a maintained holding potential of 0 mV, a number of factors can influence GABAergic currents or distort estimates thereof, including intracellular accumulation of chloride ions (Staley and Proctor, 1999; DeFazio and Hablitz, 2001; Doyon et al., 2011), depolarization-induced suppression of inhibition, voltage dependence of GABA_A_ receptors (Salin and Prince, 1996; Ransom et al., 2010), and possibly even local reverse transport of GABA by GAT-1 located in the voltage-clamped postsynaptic membrane (Minelli et al., 1995). As somatic voltage clamp recordings thus did not appear suitable to allow precise quantitative statements on the modulation of inhibition by NO-711, we focused on assessments of membrane resistance and excitability via current clamp measurements as the more promising experimental approach.

Under drug-free conditions, somatic excitability of the majority of neurons correlated positively with the degree of ongoing spontaneous activity, indicating that at the soma the excitatory impact of the mixed excitatory and inhibitory synaptic inputs dominated. When GABA uptake was impaired, this positive correlation was nullified or even inverted: during spontaneous network bursts, the neurons proved to be less excitable than in the absence of network activity, pointing to a dominance of inhibition over excitation. Outside these brief intervals of accentuated inhibition, tightly time-locked to bursts, somatic excitability and membrane resistance of cortical neurons was little affected by NO-711. This seemingly paradoxical finding suggests that submicromolar concentrations of NO-711 do not substantially enhance or induce tonic inhibitory currents: if tonic currents had been enhanced in the silent periods between bursts, the underlying hyperpolarizing or shunting conductance should have decreased somatic resistance and excitability, as is seen with high concentrations of NO-711 (Gonzalez-Burgos et al., 2009). In our experiments, such effects were clearly observed with muscimol, but were at best weak with NO-711 at the tested concentration (250 nM). The fact that some neurons even appeared hyperexcitable may be attributable to the overall strong depression of spontaneous activity by NO-711, and an according depression of hyperpolarizing conductances in the wake of the bursts, most likely Ca^2^^+^- and Na^+^-activated K^+^ currents (Compte et al., 2003; Sanchez-Vives et al., 2010).

Moreover, we reasoned that if NO-711 primarily enhanced phasic inhibition, it should modulate activity patterns in the cortical networks in a manner distinctly different from that of muscimol. Specifically, muscimol lowered the rate of network bursts (**Figure 8B**), which is likely the consequence of diminished neuronal excitability during silent periods (**Figure 7E**). By contrast, phasic inhibition was by definition restricted to network bursts; its enhancement by NO-711 was thus expected to modulate burst properties. In particular, the very efficient shortening of bursts is best explained by enhanced phasic inhibition by NO-711, in line with the observation that cortical Up states can be terminated by coordinated bouts of APs of inhibitory interneurons (Shu et al., 2003; Mann et al., 2009). We presume that the concomitant higher rate of network bursts was an indirect effect, due to a reduction of activity-dependent hyperpolarizing currents. In agreement with this notion, a mild blockade of inhibition, leading to stronger excitation, was found to have the inverse effect (Mann et al., 2009; Sanchez-Vives et al., 2010).

Finally, although the largest inhibitory current transients usually occurred at the beginning of the bursts, the brunt of the AP-depressing effect of NO-711 occurred later in the burst (**Figures 3D,E**). This observation was not made with muscimol, and is compatible with an activity-dependent, progressive enhancement of inhibition due to an extrasynaptic accumulation of GABA.

We do not rule out that tonic currents can in principle be induced or enhanced by submicromolar concentrations of NO-711. First, it must be borne in mind that tonic currents depending on synaptically released GABA may be underestimated *in vitro* compared to those *in vivo* for the simple reason that the bath perfusion potentially represents a large sink of ambient neurotransmitters (Glykys and Mody, 2007a). Second, glial cells including astrocytes tend to proliferate in organotypic cultures (del Rio et al., 1991). As GAT-1 is also found in astroglial processes, it is possible that the ratio of phasic vs. tonic currents inducible by GAT-1 impairment is distorted in organotypic cultures. These methodological issues notwithstanding, it seems that the tonic current inducible by manipulation of only GAT-1 is limited. In previous reports on cortical neurons, even complete blockade of GAT-1 with 10–100 μM NO-711, sometimes complemented with externally applied GABA, has proved to produce tonic currents which were small compared to those inducible via a combined blockade of GAT-1 and GAT-2/3 (Ulrich, 2003; Keros and Hablitz, 2005; Yamada et al., 2007; Vardya et al., 2008; Song et al., 2013). Such synergistic effects of combined GAT-1 and GAT-3 blockade were recently confirmed in a microdialysis study in rat hippocampus *in vivo* (Kersanté et al., 2013). Interestingly, in this study the EC_50_ of NO-711 for the enhancement of ambient GABA was 1100 nM, slightly above the maximal concentration tested here, and 100 nM NO-711, although *per se* clearly increasing ambient GABA, failed to show synergism with GAT-2/3 blockade (Kersanté et al., 2013), indicating that with a fraction of GAT-1 intact, spilt-over GABA may not reach sites in extracellular space harboring GAT-2/3. Furthermore, under the assumption that synaptically released GABA is the major source of ambient GABA (Bright et al., 2007; Glykys and Mody, 2007b), replenishment of ambient GABA is expected to suffer from a shortage of synaptically provided GABA under conditions of diminished neuronal activity caused by NO-711. In the light of these findings, and based on our results detailed above we conclude that NO-711 in the submicromolar concentration range (≤250 nM) depressed AP firing and modulated activity patterns primarily by enhancing phasic inhibition, with a likely contribution of spillover of GABA, and that tonic inhibition, if induced or enhanced, did not contribute substantially.

Toward higher concentrations of NO-711 (500 and 1000 nM in our study), tonic currents may come into the picture, as at these levels of GAT-1 inhibition substantial amounts of GABA would be expected to escape uptake. Particularly, GAT-1 located remote from the release sites are likely to play a role. IC_50_ values of NO-711 for GABA uptake at GAT-1 determined in glial or neuronal cell cultures range from 380 to 1238 nM, one to two orders of magnitude higher than those determined in synaptosomes (Suzdak et al., 1992; Borden, 1996; Wu et al., 2007). It is unclear why these GAT-1 should be less sensitive to NO-711; cell type- and compartment-dependent regulation by second messengers is among the possibilities (Vaz et al., 2008; Cristovao-Ferreira et al., 2009). Thus, 500–1000 nM NO-711 may have contributed to the prolongation of silent periods by enhancing tonic inhibition in our study, reminiscent of the effects of muscimol, and to the tonic currents reported in other studies by micromolar concentrations of NO-711. However, we did not investigate this concentration range in more detail.

The discussion thus far assumes that GATs remove GABA from the extracellular space. Yet, due to its operation as a passive transporter, GAT-1 can invert the direction of GABA transport, even on short time scales, depending on the membrane potential and the cross-membrane concentration gradients of GABA and the co-transported Na^+^ and Cl^-^ ions (Wu et al., 2007; Ransom et al., 2013). Likewise, based on work in acute cortical slices, the hypothesis has been put forward that GAT-2/3 may preferentially operate in reverse mode and furnish GABA to interneurons, thus constraining their activity (Kinney, 2005; Kirmse and Kirischuk, 2006). During full-fledged network bursts in our networks, a dynamic dependence of the net flux of GABA may have included brief windows of reversal, but we did not find evidence for a reverse transport of GABA over long time scales. If reverse transport had occurred on a substantial scale, impairing either GAT should have limited the amount of GABA released via this way, in other words, disinhibited the network. For NO-711, we found the opposite, namely a clear inhibition of the networks, indicative of impaired uptake as discussed above. However, we cannot exclude that GAT-2/3 blockade may have excited interneurons in the silent periods between the bursts, in which AP activity was low, and probably comparable to the low rates of spontaneous activity observed in unstimulated acute cortical slices. It is conceivable that such an effect was one source of the large variability of the effects of SNAP5114 on cortical firing rates.

### COMPARISON TO EFFECTS OF GAT-1 ANTAGONISM ON THE SYSTEMS LEVEL

GAT-1 antagonists like tiagabine or NO-711 are antiepileptics (Dalby, 2003), but it seems that the net effect of these drugs in the whole brain are not completely understood. Although *in vitro* GAT-1 antagonists can enhance inhibition to a degree comparable to that of GABA_A_ receptor modulators, *in vivo* they cause at most sedation and, at higher doses, motor impairment, but none of the more profound behavioral end points typical of GABA_A_ receptor modulators like loss of righting reflex (Suzdak et al., 1992; Smith et al., 1995; Katayama et al., 2007). Furthermore, anticonvulsant dosages of both substances were found to be far below those which measurably elevated ambient [GABA] in thalamus (Richards and Bowery, 1996). These observations collectively suggest that a measured impairment of GAT-1, as opposed to a complete blockade, is optimal for suppressing pathological activity. Based on the results of our study we propose the testable hypothesis that GAT-1 antagonists blunt pathological activity in cortical networks *in vivo* at concentrations which do not produce tonically elevated ambient [GABA], but which impose a rapidly acting, negative feedback on emerging, high-frequency AP activity via dynamically enhanced phasic inhibition.

## Conflict of Interest Statement

The authors declare that the research was conducted in the absence of any commercial or financial relationships that could be construed as a potential conflict of interest.
